# Experimental and In Silico Evaluation of Honey‐Derived Phenolic Compounds: Antioxidant Activity, Anti‐Inflammatory Potential Pharmacokinetic Properties, Toxicity Assessment, and Multitarget Anti‐Inflammatory Potential

**DOI:** 10.1155/omcl/6342617

**Published:** 2026-09-01

**Authors:** Eda Sonmez Gurer, Sevgi Durna Dastan, Zeliha Selamoglu, Ainash Oshibayeva, Taner Dastan, Nurdana Salybekova

**Affiliations:** ^1^ Department of Pharmacy, Faculty of Pharmacy, Sivas Cumhuriyet University, Sivas, Türkiye, cumhuriyet.edu.tr; ^2^ Department of Biology, Faculty of Science, Sivas Cumhuriyet University, Sivas, Türkiye, cumhuriyet.edu.tr; ^3^ Center Director, Application and Research Center, Beekeeping Development, Sivas Cumhuriyet University, Sivas, Türkiye, cumhuriyet.edu.tr; ^4^ Department of Biology, Faculty of Sciences, Khoja Akhmet Yassawi International Kazakh-Turkish University, Turkestan, Kazakhstan, iktu.kz; ^5^ Western Caspian University, Baku, Azerbaijan, wcu.edu.az; ^6^ Department of Medical Biology, Faculty of Medicine, Nigde Omer Halisdemir University, Nigde, Türkiye, ohu.edu.tr; ^7^ Department of Public Health and Scientific Research, Faculty of Medicine, Khoja Akhmet Yassawi International Kazakh-Turkish University, Turkestan, Kazakhstan, iktu.kz; ^8^ Department of Biochemistry, Faculty of Science, Sivas Cumhuriyet University, Sivas, Türkiye, cumhuriyet.edu.tr

**Keywords:** ADME, anti-inflammatory activity, antioxidant activity, Kazakhstan honey, molecular docking, phenolic compounds, toxicity prediction

## Abstract

Honey is a natural product rich in phenolic compounds that contribute to its antioxidant and anti‐inflammatory properties. The present study aimed to evaluate the biological activities of honey samples collected from different regions of Kazakhstan through experimental assays and computational analyses of major honey‐derived phenolic compounds. Ethanolic extracts of seven honey samples were prepared and assessed for extraction yield, total phenolic content, total flavonoid content (TFC), antioxidant activity using the DPPH radical scavenging assay, and anti‐inflammatory activity using the albumin denaturation inhibition assay. In addition, five representative honey‐derived phenolic compounds, namely fumaric acid, p‐hydroxybenzoic acid, p‐coumaric acid, trans‐2‐hydroxycinnamic acid, and chrysin, were subjected to comprehensive in silico analyses including ADME prediction, drug‐likeness evaluation, toxicity assessment, and multitarget molecular docking. Extraction yields ranged from 6.8% to 8.7%. The highest total phenolic content was observed in sample B12 (80.1 ± 9.7 mg GAE/g extract), whereas the highest TFC was detected in sample B10 (6.8 ± 0.5 mg quercetin equivalent (QE)/g extract). DPPH radical scavenging activity revealed IC_50_ values ranging from 2.5 ± 0.1 to 5.6 ± 0.4 mg/mL, with samples B11, B12, and B10 exhibiting the strongest antioxidant activities. In the albumin denaturation assay, all extracts demonstrated concentration‐dependent anti‐inflammatory activity, with inhibition percentages reaching 33.9% ± 2.0% at 100 mg/mL. The ADME analysis revealed that all investigated compounds exhibited favorable drug‐likeness properties and high predicted gastrointestinal (GI) absorption. Chrysin demonstrated the highest lipophilicity and receptor‐binding potential, whereas p‐coumaric acid and trans‐2‐hydroxycinnamic acid showed the most balanced pharmacokinetic profiles. Toxicity predictions performed using ProTox‐3.0, pkCSM, and admetSAR indicated low risks of hepatotoxicity and nephrotoxicity, with no predicted neurotoxicity, respiratory toxicity, carcinogenicity, or mutagenicity for any of the compounds. Molecular docking analyses against multiple inflammation‐related targets, including cyclooxygenase‐2 (COX‐2), inducible nitric oxide synthase (iNOS), nuclear factor kappa‐B (NF‐κB), tumor necrosis factor‐alpha (TNF‐α), interleukin‐6 (IL‐6), and protein kinase B (AKT1), demonstrated strong binding affinities, particularly for trans‐2‐hydroxycinnamic acid and chrysin. These compounds exhibited favorable interactions with several key mediators of inflammatory signaling, suggesting potential multitarget mechanisms of action. Computational analyses demonstrated favorable drug‐likeness characteristics and high predicted GI absorption for all investigated compounds. Toxicity predictions indicated low risks of hepatotoxicity and nephrotoxicity, with no predicted neurotoxicity, respiratory toxicity, carcinogenicity, or mutagenicity. Molecular docking analyses against multiple inflammation‐related targets, including COX‐2, iNOS, NF‐κB, TNF‐α, IL‐6, and AKT1, identified trans‐2‐hydroxycinnamic acid and chrysin as the compounds with the strongest binding affinities, suggesting their potential role as multitarget modulators of inflammatory signaling pathways. Overall, the combined experimental and computational findings demonstrate that Kazakhstan honey represents a valuable source of bioactive phenolic compounds with promising antioxidant and anti‐inflammatory properties. Among the investigated compounds, trans‐2‐hydroxycinnamic acid, p‐coumaric acid, and chrysin emerged as the most promising candidates for further pharmacological investigation. These findings provide a scientific basis for future studies exploring honey‐derived phenolics as potential complementary agents for the management of inflammatory disorders. The integration of experimental bioactivity assays with multitarget computational analyses provides novel insights into the mechanisms through which honey‐derived phenolics may contribute to the therapeutic potential of honey.

## 1. Introduction

Honey is among the oldest natural products used for medicinal purposes, with therapeutic applications documented across diverse civilizations for thousands of years [1, 2]. Beyond its established nutritional value, honey has traditionally been utilized in the management of wounds, gastrointestinal (GI) and respiratory disorders, as well as microbial infections [1]. Increasing experimental evidence has further established honey as a biologically active natural product with diverse pharmacological properties, including anti‐inflammatory, immunomodulatory, antioxidant, antimicrobial, and antiproliferative activities [2, 3]. These effects are largely associated with its complex phytochemical composition, particularly its phenolic acids, which are influenced by floral origin, geographical provenance, climatic conditions, and seasonal variation [4–6]. The phenolic fraction of honey encompasses a structurally diverse group of secondary metabolites, broadly categorized into phenolic acids and flavonoids. The phenolic acids present in honey can be broadly divided into two principal groups according to their chemical structure. The first comprises hydroxybenzoic acid derivatives, including gallic acid, p‐hydroxybenzoic acid, and vanillic acid, whereas the second encompasses hydroxycinnamic acid derivatives, such as caffeic acid, p‐coumaric acid, ferulic acid, and cinnamic acid. Honey also contains a wide range of flavonoids, including flavones such as chrysin and apigenin; flavonols such as quercetin, kaempferol, and galangin; flavanones such as pinocembrin, naringenin, and hesperetin; and flavanonols such as pinobanksin [3, 6]. Accumulating in vitro and in vivo evidence indicates that these constituents contribute substantially to the biological effects of honey by modulating oxidative stress, inflammatory signaling cascades, and immune responses [2, 3].

Oxidative stress and chronic inflammation are recognized as central mechanisms underlying the pathogenesis of numerous disorders, including cardiovascular diseases, metabolic syndrome, neurodegenerative diseases, autoimmune conditions, and respiratory inflammatory disorders [7–11]. Consequently, increasing attention has been directed toward naturally occurring polyphenols capable of simultaneously modulating multiple molecular pathways involved in inflammation and oxidative damage [12]. Unlike conventional single‐target therapeutics, phenolic compounds frequently exhibit pleiotropic biological effects by interacting with several signaling cascades, including nuclear factor kappa‐B (NF‐κB), cyclooxygenase‐2 (COX‐2), inducible nitric oxide synthase (iNOS), tumor necrosis factor‐alpha (TNF‐α), interleukin‐6 (IL‐6), and phosphoinositide 3‐kinase/protein kinase B (PI3K/Akt) pathways [7–11, 13–18]. This multitarget mode of action has generated considerable interest in their potential application as complementary therapeutic agents for inflammatory diseases. Honey has a long‐standing history of use in traditional medicine for alleviating respiratory symptoms, particularly cough and conditions such as bronchitis and asthma [19, 20]. Although its traditional respiratory applications have often been attributed to demulcent and mucolytic effects, emerging evidence indicates that honey‐derived phenolic constituents may also influence airway inflammatory processes through antioxidant and immunomodulatory mechanisms [12]. Experimental investigations have further demonstrated that selected phenolic acids and flavonoids can modulate key features of allergic airway inflammation, including the production of pro‐inflammatory cytokines, mast cell degranulation, eosinophilic infiltration, and excessive mucus secretion [7–11, 17, 18]. Collectively, these findings provide a mechanistic basis for proposing honey‐derived phenolics as potential bioactive modulators of airway inflammatory responses, while further experimental and clinical studies are required to establish their therapeutic relevance.

Among the honey‐producing regions of the world, Central Asia remains relatively underexplored despite its rich ecological diversity. Kazakhstan possesses extensive steppe, semi‐arid, and mountainous ecosystems that support a unique melliferous flora capable of influencing honey composition. Recent chemical analyses of honey samples collected from southern Kazakhstan identified several phenolic compounds, including fumaric acid, p‐hydroxybenzoic acid, p‐coumaric acid, trans‐2‐hydroxycinnamic acid, and chrysin, as recurring constituents across multiple geographical locations [19]. Although these compounds have individually been reported in different honey types worldwide [3, 21], comprehensive evaluations integrating their biological activities, pharmacokinetic behavior, toxicity profiles, and molecular interaction patterns remain limited.

In parallel with advances in experimental natural product research, computational approaches have become indispensable tools for the early‐stage evaluation of bioactive molecules. In silico platforms such as SwissADME facilitate the prediction of physicochemical properties, oral bioavailability, drug‐likeness, pharmacokinetic behavior, and toxicity, thereby enabling the prioritization of compounds for further pharmacological investigation [22–24]. Furthermore, molecular docking techniques provide valuable insights into the potential interactions of natural compounds with disease‐related molecular targets, allowing the identification of possible mechanisms underlying their biological activities.

Therefore, the present study combined experimental and computational approaches to comprehensively evaluate the biological potential of honey samples collected from different regions of Kazakhstan. Ethanolic honey extracts were first assessed for extraction yield, total phenolic content, total flavonoid content (TFC), antioxidant activity using the DPPH radical scavenging assay, and anti‐inflammatory activity using the albumin denaturation inhibition assay. Subsequently, five representative honey‐derived phenolic compounds—fumaric acid, p‐hydroxybenzoic acid, p‐coumaric acid, trans‐2‐hydroxycinnamic acid, and chrysin—were subjected to integrated in silico analyses, including physicochemical characterization, ADME prediction, drug‐likeness evaluation, toxicity assessment, and molecular docking against multiple inflammation‐related targets. By combining experimental bioactivity data with computational pharmacological analyses, this study aimed to identify promising honey‐derived phenolic compounds and to provide mechanistic insights into their potential roles in the management of inflammatory disorders.

## 2. Material and Methods

### 2.1. Honey Samples and Compound Selection

Honey samples were collected from distinct localities of Kazakhstan (Table 1): Shauldir, Kogam Village, Otrar District, Turkestan Region, Kyzylorda Region, Shymkent, and Shieli District. These samples were subjected to compositional analysis and biological activity assessment as part of a broader investigation into the bioactive constituents of regionally sourced honeys [19]. Based on the comprehensive review of the existing literature on honey composition, some active compounds that were commonly identified across the samples were selected for in silico evaluation. The selection of the phenolic compounds was based on their consistent detection across multiple honey samples obtained from different geographical locations in Kazakhstan, as reported in the referenced analytical study [19]. In this context, these compounds are selected according to their relative abundance. Other phenolic constituents reported in honey were not included in the present study because they were either detected only in specific samples, occurred at trace levels, or lacked sufficient consistency across the dataset to support a comparative in silico evaluation. Therefore, the selected compounds represent a subset of structurally diverse and widely distributed phenolic constituents suitable for preliminary pharmacokinetic and drug‐likeness screening.

**Table 1:** Geographical origins of honey samples collected from Kazakhstan.

Sample code	Location
B1	Shauldir
B2	Kelintobe village
B8	Shieli district
B10	Kyzylorda region
B11	Kogam village
B12	Otrar district
B13	Turkestan region

### 2.2. In Silico Assessment

The simplified molecular input line entry system (SMILES) notations for each compound were retrieved from the PubChem database (National Center for Biotechnology Information, U.S. National Library of Medicine) [24]. The SMILES notations of compounds are presented as follows: C( = C/C( = O)O)\C( = O) for fumaric acid; C1 = CC( = CC = C1C( = O)O)O for p‐hydroxybenzoic acid; C1 = CC( = CC = C1/C = C/C( = O)O)O for p‐coumaric acid; C1 = CC = C(C( = C1)/C = C/C( = O)O)O for trans‐2‐hydroxycinnamic acid; and last C1 = CC = C(C = C1)C2 = CC( = O)C3 = C(C = C(C = C3O2)O)O for chrysin. The physicochemical and pharmacokinetic properties of the compounds were evaluated using the SwissADME web platform (http://www.swissadme.ch), a freely accessible computational tool developed by the Swiss Institute of Bioinformatics [23–25]. The toxicity parameters of the compounds, including LD_50_ values, were determined using the ProTox‐3.0 online server [26]. The Brain or IntestinaL EstimateD permeation (BOILED‐Egg) model was employed to visualize predicted based on topological polar surface area (TPSA) coordinates [25].

### 2.3. The Docking Analysis

The ligands selected for this study, namely, fumaric acid, p‐hydroxybenzoic acid, p‐coumaric acid, trans‐2‐hydroxycinnamic acid, and chrysin, were retrieved from the PubChem database in structure data file (.sdf) format [24] with the following PubChem Compound Identifiers (CIDs): 444972, 46235485, 637542, 155489622, and 5281607, respectively. To obtain the thermodynamically most stable conformations, the three‐dimensional (3D) structures of the compounds were subjected to geometry optimization and energy minimization using Avogadro software (Version 1.2) [27]. The optimization process was performed employing the Merck Molecular Force Field (MMFF94), and the minimized structures were subsequently converted into formats suitable for molecular docking analyses. Target proteins were retrieved from the UniProt database [28]. The selected targets included COX‐2 (UniProt: P35354), iNOS (UniProt: P35228), NF‐κB p65 subunit (UniProt: Q04206), TNF‐α (UniProt: P01375), IL‐6 (UniProt: P05231), and RAC‐alpha serine/threonine‐protein kinase (AKT1; UniProt: P31749). The corresponding 3D protein structures were obtained from the Research Collaboratory for Structural Bioinformatics Protein Data Bank (RCSB PDB) [29]. When experimentally resolved crystal structures were unavailable, predicted structures were retrieved from the AlphaFold Protein Structure Database [30]. All protein structures were prepared and processed prior to the docking analyses.

The binding affinities and potential interaction mechanisms between the optimized ligands and target proteins were evaluated using the CB‐Dock2 web server [31]. CB‐Dock2 is an automated molecular docking platform that identifies potential binding cavities on the protein surface and performs blind docking using the AutoDock Vina docking engine [32]. The prepared ligand and receptor files were uploaded to the server, and the most favorable binding cavities were automatically detected based on cavity volume and docking score. For each ligand–protein complex, the lowest binding energy value (Vina score, kcal/mol) was recorded, and the most stable binding conformations were selected for further analysis.

To characterize the molecular interactions underlying ligand binding, the top‐ranked protein–ligand complexes obtained from the docking analyses were visualized using the BIOVIA Discovery Studio Visualizer 2024 [33]. Two‐dimensional (2D) interaction diagrams were generated to identify and evaluate key interactions between the ligands and amino acid residues within the active sites of the target proteins. These interactions included hydrogen bonds, hydrophobic interactions (π–π stacking, π–alkyl interactions, and alkyl contacts), van der Waals forces, and electrostatic interactions. The interaction profiles were comprehensively analyzed and used to interpret the binding mechanisms of the investigated compounds.

To validate the molecular docking protocol, a redocking procedure was performed using the cocrystallized ligand retrieved from each protein crystal structure. The native ligand was first removed from the binding site and then redocked into the same active site using the identical Glide XP docking protocol applied to all investigated compounds. The predicted binding pose was superimposed on the experimentally determined crystallographic conformation, and the root mean square deviation (RMSD) was calculated. An RMSD value below 2.0 Å was considered indicative of a reliable docking protocol and accurate reproduction of the experimental binding mode.

### 2.4. Extraction of Bee Products

Honey samples were obtained from local beekeepers in Southern Kazakhstan. The collected samples were stored at 4°C until analysis. For extraction, 5 g of each sample was mixed with 100 mL of 70% ethanol and incubated in a shaker incubator at room temperature for 48 h. The mixtures were filtered using Whatman No. 1 filter paper and concentrated under reduced pressure using a rotary evaporator at 40°C. The obtained crude extracts were stored at −20°C until further analyses. Stock solutions were prepared in dimethyl sulfoxide (DMSO) before biological assays.

### 2.5. Determination of TFC

TFC was determined using an aluminum chloride colorimetric assay. Briefly, 0.5 mL of the extract was mixed with 0.5 mL of 2% aluminum chloride solution and incubated for 30 min at room temperature. The absorbance was measured at 415 nm. Quercetin was used for calibration, and the results were expressed as mg quercetin equivalents (QE)/g extract [34].

### 2.6. Determination of Total Phenolic Content

Total phenolic content was determined according to the Folin–Ciocalteu method by Singleton et al. [35] with minor modifications for microplate analysis. Briefly, 20 µL of extract solution was mixed with 100 µL of 10% Folin–Ciocalteu reagent and incubated for 5 min. Subsequently, 80 µL of 7.5% sodium carbonate solution was added, and the reaction mixture was incubated in the dark for 30 min at room temperature. Absorbance was measured at 765 nm using a BioTek Epoch microplate spectrophotometer. Gallic acid was used for the preparation of the calibration curve, and the results were expressed as mg GAE/g extract. A strong linear relationship was observed between absorbance and concentration, yielding the regression equation *y* = 0.978*x* + 0.256. The total phenolic content of the honey extracts was calculated from the calibration curve and expressed as milligrams of gallic acid equivalents per gram of extract.

### 2.7. DPPH Radical Scavenging Assay

The antioxidant activity of the extracts was evaluated using the DPPH free‐radical scavenging assay [36]. Different concentrations of extracts (10–500 µg/mL) were prepared and mixed with a 0.1 mM DPPH solution. The reaction mixtures were incubated in the dark for 30 min at room temperature. Absorbance was measured at 517 nm using a microplate reader. The radical scavenging activity was calculated as percentage inhibition relative to the control group, and IC50 values were determined. The commercially available standard quercetin (Sigma–Aldrich) was used as a reference compound in all activity assays for comparative analysis.

### 2.8. Anti‐Inflammatory Activity Assay

The anti‐inflammatory potential of the alcoholic extracts of the tested bee product samples was evaluated based on their ability to inhibit heat‐induced denaturation of bovine serum albumin (BSA). The reduction in protein denaturation indicates anti‐inflammatory activity. Briefly, 0.5 mL of 1% BSA solution was mixed with 0.1 mL of the test samples at different concentrations. The pH of the reaction mixture was adjusted to 6.3 using PBS. The mixtures were incubated at 37°C for 20 min, followed by heating at 70°C for 5–10 min to induce protein denaturation. After incubation, the mixtures were cooled to room temperature. The resulting turbidity was measured spectrophotometrically at 560 nm [37]. The commercially available standard quercetin (Sigma–Aldrich) was used for comparative analysis as a standard pure drug molecule. The higher percentage of inhibition indicates the stronger anti‐inflammatory activity of the tested compounds. The percentage inhibition of protein denaturation was calculated using the following formula:

%Inhibition= Absorbance of control −absorbance of sample:absorbance of control ×100.

### 2.9. Statistical Analysis

All experiments were performed in triplicate, and results were expressed as mean ± standard deviation (SD). Statistical analyses were carried out using GraphPad Prism software. Statistical significance was considered at *p* < 0.05.

## 3. Results and Discussion

### 3.1. Extraction Yield of Honey Samples

Extractions of honey samples were prepared using a 70% ethanol solvent. The yields of the obtained extracts were calculated as percentages (Table 2).

**Table 2:** Percentage yields of honey extracts.

Sample code	Location	Yield (%)
B1	Shauldir	6.9
B2	Kelintobe village	7.4
B8	Shieli district	7.9
B10	Kyzylorda region	6.8
B11	Kogam village	7.8
B12	Otrar district	8.3
B13	Turkestan region	8.7

### 3.2. The DPPH• Radical Scavenging Activity and Total Flavonoid and Phenolic Contents

The total flavonoid and phenolic content of all extracts are given in Table 3. It was observed that the highest total phenolic content was in sample B12 (80.1 ± 9.7 mg GAE/g) and the lowest was in sample B1 (33.9 ± 6.7 mg GAE/g). The highest TFC was observed in sample B10 (6.8 ± 0.5 mg QE/g) and the lowest in sample B1 (4.5 ± 0.4 mg QE/g); the total phenolics and flavonoid contents of all extracts are presented in Table 3. Samples B10, B11, and B12 are the extracts with high antioxidant capacity. IC50 represents the amount of a substance required to demonstrate its antioxidant effect. Low IC50 values indicate high antioxidant activity, whereas high IC50 values indicate low antioxidant activity. According to Table 3, the IC50 values of the honey extracts range from 2.5 to 5.6 mg/mL. Based on the IC50 values of the ethanol extracts of the honey samples, the ranking of DPPH• radical scavenging activity—from lowest to highest—was found to be B13 < B1 < B8 < B2 < B010 < B12 < B11 < ascorbic acid. The IC50 values of all extracts were found to be significantly higher than those of ascorbic acid (Table 3).

**Table 3:** Total flavonoid–phenolics content and DPPH• radical scavenging activity of ethanolic extracts of honey.

Sample code	Total flavonoid content (mg QE/g extract ± SD)	Total phenolic content (mg GAE/g extract ±SD)	DPPH• radical scavenging activity IC50 (mg/mL)
B1	4.5 ± 0.4	33.9 ± 6.7	4.1 ± 0.3
B2	4.7 ± 0.3	40.7 ± 7.4	3.5 ± 0.2
B8	6.1 ± 0.7	70.3 ± 10.2	3.7 ± 0.2
B10	6.8 ± 0.5	76.9 ± 10.7	2.9 ± 0.2
B11	5.5 ± 0.4	43.9 ± 6.5	2.5 ± 0.1
B12	6.7 ± 0.6	80.1 ± 9.7	2.8 ± 0.2
B13	5.7 ± 0.5	61.3 ± 7.1	5.6 ± 0.4
Ascorbic acid	—	—	0.5 ± 0.1

### 3.3. Anti‐Inflammatory Activity of Honey Extracts

The albumin denaturation inhibition activities of ethanol extracts of honey samples collected from different localities in Kazakhstan are presented in Table 4. The albumin denaturation inhibition test is a reliable and widely used in vitro method for the preliminary screening of anti‐inflammatory activity. Protein denaturation plays a significant role in inflammatory processes and constitutes one of the mechanisms of action of anti‐inflammatory drugs. Therefore, it is thought that extracts showing high activity in this test may also have the potential for anti‐inflammatory effects under in vivo conditions. In conclusion, ethanol extracts of honey samples collected from Kazakhstan exhibited significant anti‐inflammatory activity in the albumin denaturation inhibition test. Further studies are recommended to isolate and characterize the active compounds, apply different in vitro anti‐inflammatory tests, and evaluate their in vivo activity.

**Table 4:** Percentage of albumin denaturation inhibition of ethanolic extracts of honey.

Sample code	100 mg/mL (%)	10 mg/mL (%)	1 mg/mL (%)
B1	33.9 ± 2.0	30.5 ± 3.6	25.1 ± 3.1
B2	23.8 ± 2.5	21.4 ± 3.8	17.4 ± 1.1
B8	27.5 ± 2.3	25.4 ± 3.9	19.3 ± 2.3
B10	29.1 ± 2.7	25.7 ± 3.6	18.4 ± 2.4
B11	25.9 ± 2.2	22.5 ± 3.8	20.2 ± 2.2
B12	29.3 ± 2.4	27.6 ± 3.9	21.3 ± 2.4
B13	29.4 ± 2.6	26.3 ± 3.9	22.5 ± 2.5
Diclofenac sodium^a^ (standard inhibitor and positive control)	80.3 ± 7.7	66.8 ± 3.8	60.6 ± 3.5

^a^Standard inhibitor for albumin denaturation test.

### 3.4. Estimated Physicochemistry and Pharmacokinetic Structures of Compounds

The molecular structures of five active components identified in Kazakhstani honey are shown in Table 5.

**Table 5:** 2D structures and bioavailability radar plot of some common compounds found in Kazakhstani honey. (2D structure created in https://BioRender.com).

2D structure	Bioavailability radar
Fumaric acid 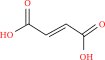	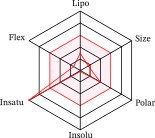
p‐Hydroxybenzoic acid 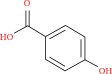	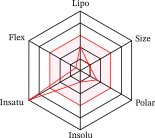
p‐Coumaric acid 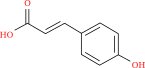	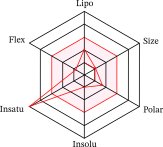
trans‐2‐Hydroxycinnamic acid 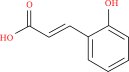	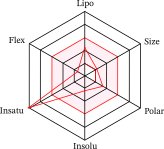
Chrysin 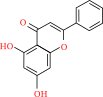	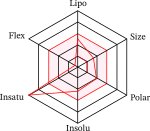

**Figure 1:**
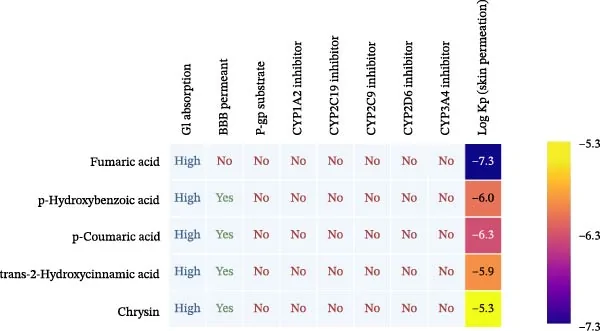
Pharmacokinetic properties of chemical compounds (created in https://BioRender.com).

The physicochemical properties of the investigated compounds were obtained from SwissADME [38]. Chrysin displayed the highest number of aromatic heavy atoms and molar refractivity, suggesting greater potential for π–π stacking and van der Waals interactions with biological macromolecules, features generally linked to stronger receptor binding and molecular recognition [39]. The TPSA values ranged from 57.5 to 74.6 Å^2^ [40], remaining within the range generally considered favorable for passive membrane diffusion. Furthermore, all compounds complied with the hydrogen‐bond donor and acceptor requirements of Lipinski’s Rule of Five, indicating a balanced physicochemical profile that supports intermolecular interactions while maintaining membrane permeability [23, 38]. Notably, p‐coumaric acid and trans‐2‐hydroxycinnamic acid, despite sharing an identical molecular formula and weight (164.6 g/mol), differ in hydroxyl‐substituent position; this positional isomerism may affect intramolecular hydrogen bonding, conformational flexibility, and target recognition, and could account for divergent biological activities [41]. Collectively, the physicochemical assessment suggests that chrysin and the hydroxycinnamic acid derivatives possess structural characteristics that are particularly favorable for interaction with biological targets. Lipophilicity represents a key determinant of membrane permeability, oral bioavailability, tissue distribution, and protein‐binding behavior during drug development [42]. As shown in Table 5, chrysin exhibited the highest consensus Log *p* value (2.5) among the evaluated compounds. This observation is consistent with its extended aromatic framework and relatively low polar surface area compared with its molecular size. Such a lipophilicity profile may facilitate passive transcellular transport and promote hydrophobic interactions within the binding pockets of target proteins. The pharmacokinetic predictions further demonstrated that all investigated compounds possess high GI absorption, indicating favorable oral bioavailability potential. With the exception of fumaric acid, all compounds were predicted to permeate the blood–brain barrier (BBB), suggesting the possibility of systemic distribution to central nervous system tissues. In addition, none of the molecules were identified as P‐glycoprotein (P‐gp) substrates or significant cytochrome P450 (CYP450) enzyme inhibitors, implying a low propensity for transporter‐mediated efflux and a reduced risk of metabolic or drug–drug interactions. Skin permeability analysis revealed that chrysin exhibited the highest predicted permeability among the evaluated compounds, further supporting its favorable pharmacokinetic profile. Overall, the integrated physicochemical and ADME analyses indicate that chrysin, together with the hydroxycinnamic acid derivatives, demonstrates the most advantageous pharmacokinetic and drug‐likeness characteristics, whereas fumaric acid appears to possess a comparatively more restricted pharmacokinetic profile (Figure 1).

The predicted aqueous solubility profiles of the investigated compounds, obtained from SwissADME. According to the information obtained from the SwissADME database, predicted aqueous solubility profiles are presented. Fumaric acid exhibited the highest aqueous solubility among the evaluated compounds, consistent with its low molecular weight, high polarity, and lack of hydrophobic aromatic moieties—properties that favor dissolution in aqueous media. Chrysin, conversely, showed the lowest predicted solubility, while the remaining compounds were classified as soluble, indicating generally favorable oral dissolution characteristics [43]. Chrysin’s limited aqueous solubility can be attributed to the extensive aromatic conjugation of its flavone backbone together with its comparatively higher lipophilicity. Although chrysin displayed the most favorable membrane permeability and target‐binding potential, its poor solubility represents a notable pharmaceutical limitation; formulation strategies such as nanoencapsulation, cyclodextrin complexation, or solid dispersion systems may therefore be warranted to enhance its dissolution rate and oral bioavailability [44, 45]. Pharmacokinetic predictions further indicated high GI absorption potential for all five compounds, consistent with their compliance with Lipinski’s Rule of Five and favorable TPSA values. These findings support the likelihood of efficient intestinal uptake following oral administration. Regarding BBB permeability, all compounds except fumaric acid were predicted to penetrate the BBB, indicating differing capacities for central nervous system distribution [25]. An additional noteworthy finding, obtained from SwissADME, was that none of the investigated compounds were predicted to act as P‐gp substrates, nor were they identified as inhibitors of the major cytochrome P450 (CYP450) isoforms [46]. These properties are particularly relevant from a pharmacokinetic perspective as P‐gp‐mediated efflux and CYP450‐mediated metabolism represent major determinants of drug absorption, distribution, and elimination. The absence of P‐gp substrate characteristics suggests that intracellular concentrations of these compounds are unlikely to be substantially reduced through active efflux mechanisms at biological barriers, including the intestinal epithelium and the BBB [46].

Likewise, the lack of predicted inhibitory activity toward major CYP450 enzymes indicates a potentially low risk of metabolic interference and drug–drug interactions [46]. This observation may be particularly important in the context of respiratory diseases, where patients frequently receive multiple pharmacological agents. Several therapeutic classes used in asthma management, including corticosteroids, leukotriene receptor antagonists, and certain immunomodulatory agents, undergo metabolism through CYP3A4‐ and CYP2D6‐associated pathways. Therefore, the predicted absence of CYP450 inhibition by the investigated honey‐derived phenolic compounds suggests a favorable pharmacokinetic profile and supports their potential suitability for adjunctive use in polypharmacy settings [47]. Overall, the combined solubility and ADME analyses indicate that while chrysin exhibits the most advantageous membrane permeability and target‐interaction characteristics, its limited aqueous solubility may constrain its pharmaceutical applicability. Conversely, the hydroxycinnamic acid derivatives appear to offer a more balanced physicochemical and pharmacokinetic profile, characterized by favorable solubility, high GI absorption, and a low predicted risk of transporter‐ or enzyme‐mediated interactions.

The drug‐likeness analysis, based on five complementary predictive models, demonstrated that all investigated compounds possess characteristics consistent with orally active molecules. None of the compounds violated the Lipinski, Veber, or Egan rules, suggesting an overall favorable balance between molecular size, polarity, lipophilicity, and flexibility [38, 40, 48, 49]. Notably, chrysin emerged as the most promising candidate from a structural perspective as it was the only molecule to satisfy all five drug‐likeness filters without any violations. Nevertheless, its predicted bioavailability score (0.55) was lower than that of the remaining compounds (0.85), highlighting the importance of considering solubility‐related limitations alongside conventional drug‐likeness metrics. This finding suggests that although chrysin exhibits highly favorable molecular features, formulation strategies aimed at improving its aqueous solubility may be necessary to fully realize its pharmacological potential [44].

The pharmacokinetic and drug‐likeness characteristics of the selected compounds were predicted using the SwissADME web‐based platform [25]. The BOILED‐Egg analysis indicated that all five compounds fell within the white area of the model, suggesting a favorable probability of passive GI absorption. Most of the compounds were also predicted to occur within the yolk region, which is associated with a higher likelihood of passive BBB permeation. Fumaric acid was the only compound predicted to remain outside the yolk, a finding that may be related to its relatively high polarity and TPSA together with its comparatively low lipophilicity. The predicted BBB permeability should, however, be interpreted as a pharmacokinetic characteristic rather than evidence of biological or therapeutic activity. Although penetration into the central nervous system may be relevant when considering potential interactions between neuroinflammatory and immune processes, the present findings are derived exclusively from computational predictions. The central nervous system and immune system are known to communicate bidirectionally, and this interaction can influence inflammatory processes through neurogenic inflammation and immune‐mediated signaling mechanisms [7–11]. Accordingly, the predicted BBB permeability of chrysin and the hydroxycinnamic acid derivatives may indicate the possibility of activity in both peripheral and central compartments. Nevertheless, this interpretation remains hypothetical and should be confirmed through appropriate experimental studies. Therefore, the BBB permeability predictions reported herein should primarily be considered as indicators of pharmacokinetic behavior rather than direct evidence of therapeutic efficacy. The bioavailability radar plots presented provide a comprehensive visualization of six key physicochemical properties associated with oral bioavailability. Among the investigated compounds, p‐coumaric acid and trans‐2‐hydroxycinnamic acid exhibited the most balanced profiles with respect to lipophilicity, molecular size, polarity, solubility, flexibility, and saturation, all of which fall within the optimal ranges proposed for orally active molecules [23]. Taken together, the integrated evaluation of physicochemical characteristics, pharmacokinetic parameters, and drug‐likeness properties identified p‐coumaric acid and trans‐2‐hydroxycinnamic acid as the most promising candidates in terms of predicted oral bioavailability, followed by p‐hydroxybenzoic acid, chrysin, and fumaric acid.

### 3.5. Oral Toxicity Prediction

The toxicological properties of the selected compounds were assessed using the ProTox‐3.0 online platform, a widely applied computational tool for predicting the toxicity of chemical substances. The predicted median lethal dose (LD_50_) values varied among the investigated compounds. Chrysin exhibited the highest LD_50_ value (3919 mg/kg), indicating the lowest predicted acute oral toxicity. In contrast, fumaric acid showed the lowest LD_50_ value (1350 mg/kg). The predicted LD_50_ values for p‐hydroxybenzoic acid, p‐coumaric acid, and trans‐2‐hydroxycinnamic acid were 2200, 2850, and 2850 mg/kg, respectively. In addition to LD_50_ estimation, toxicity classes were determined using the ProTox‐II classification system, which categorizes compounds into six toxicity classes, ranging from the most toxic (Class 1) to the least toxic (Class 6). According to these predictions, all investigated bioactive compounds were assigned to Toxicity Class 5, indicating a relatively low level of acute oral toxicity. Collectively, these findings suggest that the selected honey‐derived phenolic compounds possess favorable safety profiles and may be considered promising candidates for further pharmacological investigation. However, as these assessments are based exclusively on computational models, additional in vitro and in vivo toxicological studies are required to confirm their safety and biological relevance.

### 3.6. Molecular Docking Result

**Figure 2:**
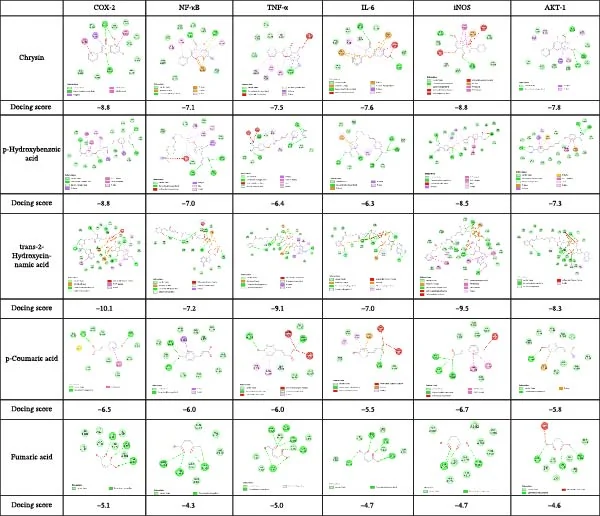
Molecular docking result.

When the molecular docking scores were evaluated, marked differences in binding affinities among the compounds toward the target proteins became evident. Overall, all the docking scores and 2D interaction diagrams are given in Figure 2. Among the five investigated compounds, trans‐2‐hydroxycinnamic acid exhibited the strongest binding affinities across the selected inflammatory targets. Notably, it demonstrated the highest docking scores against COX‐2 (−10.1 kcal/mol), iNOS (−9.5 kcal/mol), and TNF‐α (−9.1 kcal/mol), suggesting a strong interaction potential with key mediators involved in inflammatory processes. Furthermore, favorable binding affinities were also observed for NF‐κB (−7.2 kcal/mol), IL‐6 (−7.0 kcal/mol), and AKT‐1 (−8.3 kcal/mol), indicating that this compound may exert its biological activity through the simultaneous modulation of multiple inflammation‐related signaling pathways. Chrysin emerged as the second most promising compound, displaying strong binding affinities toward COX‐2 (−8.8 kcal/mol) and iNOS (−8.8 kcal/mol), as well as notable interactions with TNF‐α (−7.5 kcal/mol), IL‐6 (−7.6 kcal/mol), and AKT‐1 (−7.8 kcal/mol). Analysis of the ligand–protein interaction profiles revealed the presence of multiple hydrogen bonds and hydrophobic interactions, which may contribute to the stability of the predicted binding complexes. These findings are consistent with previous reports describing the anti‐inflammatory and antioxidant properties of chrysin. p‐Hydroxybenzoic acid also demonstrated considerable binding potential, particularly against COX‐2 (−8.8 kcal/mol) and iNOS (−8.5 kcal/mol). However, its interactions with TNF‐α (−6.4 kcal/mol) and IL‐6 (−6.3 kcal/mol) were comparatively weaker. This binding profile suggests that the anti‐inflammatory effects of p‐hydroxybenzoic acid may be more closely associated with the modulation of enzymatic inflammatory mediators than cytokine‐related targets. In contrast, p‐coumaric acid exhibited moderate binding affinities toward all investigated proteins. The strongest interactions were observed with COX‐2 (−6.5 kcal/mol) and iNOS (−6.7 kcal/mol), whereas the docking scores for the remaining targets ranged from −5.5 to −6.0 kcal/mol. Although these values indicate a measurable interaction potential, the binding affinities were generally lower than those observed for trans‐2‐hydroxycinnamic acid, chrysin, and p‐hydroxybenzoic acid. Fumaric acid displayed the weakest binding affinities among all the tested compounds. Docking scores ranged from −4.3 to −5.1 kcal/mol across all target proteins, suggesting relatively limited interaction strength. Therefore, its direct inhibitory potential against the investigated inflammatory targets appears to be lower than that of the other phenolic compounds evaluated in this study. Overall, the molecular docking results revealed the following ranking of binding affinity: trans‐2‐hydroxycinnamic acid > chrysin ≈ p‐hydroxybenzoic acid > p‐coumaric acid > fumaric acid.

Prior to docking simulations, the docking protocol was validated through a redocking procedure. The cocrystallized ligand was successfully reproduced within the binding pocket, yielding an RMSD value of 0.87 Å. Since the obtained RMSD value was well below the commonly accepted threshold of 2.0 Å, the docking protocol was considered reliable for subsequent docking analyses of the investigated phenolic compounds.

### 3.7. Evaluation of Inflammatory Signaling Pathways

Among the investigated compounds, p‐coumaric acid has been extensively associated with anti‐inflammatory activity through the downregulation of COX‐2 and iNOS expression via the inhibition of the NF‐κB and MAPK signaling pathways. Furthermore, it has been shown to reduce the production of key pro‐inflammatory cytokines, including TNF‐α and IL‐6, in both in vitro and in vivo inflammatory models [14, 15]. The favorable pharmacokinetic characteristics identified in the present study, particularly its balanced lipophilicity and high predicted GI absorption, support the biological plausibility of these reported effects following oral administration. Likewise, trans‐2‐hydroxycinnamic acid has been reported to exhibit antioxidant and anti‐inflammatory properties through the modulation of inflammatory signaling cascades [16]. The similarity of its predicted ADME profile to that of p‐coumaric acid further supports its potential as a bioactive compound capable of influencing multiple inflammatory pathways. Fumaric acid and its derivatives, particularly dimethyl fumarate (DMF), have been reported to activate the nuclear factor erythroid 2‐related factor 2 (Nrf2) signaling pathway, thereby enhancing cellular antioxidant defense mechanisms while simultaneously suppressing NF‐κB‐mediated production of pro‐inflammatory cytokines [13]. This dual mechanism highlights the potential role of fumarate‐derived compounds in regulating oxidative stress and inflammation. Chrysin deserves particular attention owing to its well‐documented ability to suppress NF‐κB signaling, regulate mast cell activation, and attenuate IgE‐mediated immune responses, mechanisms that are highly relevant to chronic inflammatory and allergic disorders [17, 18]. Consistent with these observations, the present study identified chrysin as the compound possessing the highest lipophilicity and molar refractivity values, characteristics that may facilitate receptor binding and membrane permeation. Nevertheless, its relatively poor aqueous solubility represents a major pharmacokinetic limitation that could restrict its systemic bioavailability. In this regard, formulation strategies such as nanoencapsulation, cyclodextrin inclusion complexes, and other advanced drug‐delivery approaches have been proposed to overcome this limitation and improve its therapeutic applicability [44, 45].

In addition, p‐hydroxybenzoic acid has been reported to exert antioxidant activity through direct free‐radical scavenging mechanisms associated with its phenolic hydroxyl group. Experimental studies have demonstrated its ability to neutralize reactive oxygen species, including superoxide radicals, while structurally related hydroxybenzoic acid derivatives have also been shown to suppress the expression of pro‐inflammatory mediators [41, 42]. The considerable diversity in the physicochemical properties of the investigated compounds, ranging from the highly hydrophilic fumaric acid to the comparatively lipophilic chrysin, suggests distinct biodistribution patterns and complementary biological functions. While fumaric acid may preferentially distribute within aqueous compartments and primarily contribute to the activation of Nrf2‐mediated antioxidant pathways, chrysin and the hydroxycinnamic acid derivatives may more readily access intracellular molecular targets through passive membrane permeation. Such complementary pharmacological behavior may partially explain the long‐recognized observation that whole honey frequently exhibits biological activities that exceed those of its isolated constituents [2, 20]. However, it should be emphasized that the present computational analyses do not permit the evaluation of synergistic or additive interactions among individual compounds. Experimental validation using combination studies in relevant cellular and animal models of inflammation will therefore be required to verify this hypothesis.

Taken together, the ADME, drug‐likeness, and molecular docking analyses suggest that p‐coumaric acid, trans‐2‐hydroxycinnamic acid, and chrysin possess the most favorable pharmacokinetic and mechanistic characteristics among the compounds evaluated. Their predicted ability to interact with multiple inflammation‐related targets, combined with favorable oral absorption profiles and natural origin, supports their consideration as promising candidates for complementary or adjunctive therapeutic strategies in inflammatory diseases. The observed multitarget interaction patterns further indicate that these compounds may be suitable for future investigation within integrated therapeutic approaches designed to simultaneously modulate several inflammatory signaling pathways. Several limitations of the present study should nevertheless be acknowledged. The SwissADME platform relies on predictive computational algorithms, which, although extensively validated, cannot fully replicate the complexity of in vivo pharmacokinetics, including first‐pass hepatic metabolism, microbiome‐mediated biotransformation, plasma protein binding, and tissue‐specific accumulation processes [22]. Similarly, the BOILED‐Egg model predicts BBB permeability primarily on the basis of passive diffusion and does not account for active transport systems, receptor‐mediated transcytosis, or transporter‐mediated efflux mechanisms beyond P‐gp. Moreover, the drug‐likeness filters applied in this study were originally developed using datasets dominated by synthetic pharmaceutical compounds, which may limit their applicability to naturally occurring low‐molecular‐weight phenolic compounds. This limitation is reflected by the Muegge filter violations observed for certain compounds with molecular weights below 200 g/mol.

Because safety assessment represents a critical step in the development of bioactive molecules for pharmaceutical and nutraceutical applications, additional toxicity endpoints were evaluated using the pkCSM, admetSAR, and ProTox‐3.0 platforms. The toxicity predictions indicated that fumaric acid, p‐hydroxybenzoic acid, p‐coumaric acid, trans‐2‐hydroxycinnamic acid, and chrysin exhibit low predicted risks of hepatotoxicity and nephrotoxicity, suggesting a low probability of adverse effects on the liver and kidney function. Furthermore, none of the investigated compounds demonstrated predicted neurotoxicity, respiratory toxicity, carcinogenicity, or mutagenicity, indicating the absence of major organ‐specific or genotoxic safety concerns. No substantial differences were observed among the compounds, as all exhibited similarly favorable toxicological profiles. Collectively, these findings suggest that the investigated phenolic compounds combine promising biological activity with a potentially favorable safety profile, supporting their further evaluation as pharmacological or nutraceutical candidates. Nevertheless, these conclusions are derived exclusively from in silico prediction models and should therefore be confirmed through comprehensive in vitro and in vivo toxicological investigations. The consistently low predicted toxicity risks observed across the evaluated compounds further support their potential suitability for future preclinical development.

Collectively, these findings suggest that trans‐2‐hydroxycinnamic acid and chrysin possess the most favorable interaction profiles with multiple inflammation‐related targets, including COX‐2, NF‐κB, TNF‐α, IL‐6, iNOS, and AKT‐1. Their ability to interact with several key regulators of inflammatory signaling supports the hypothesis that these compounds may contribute substantially to the anti‐inflammatory activity of honey through a multitarget mechanism of action. Nevertheless, further experimental studies are required to validate the biological relevance of these computational predictions. The expanded molecular docking analysis demonstrated that the investigated phenolic compounds interact with multiple inflammation‐related proteins rather than a single enzymatic target. Particularly, chrysin exhibited consistently strong binding affinities across several key regulators of inflammatory signaling, supporting previous experimental evidence suggesting the modulation of the NF‐κB‐associated inflammatory network. These findings strengthen the mechanistic relevance of the computational analyses and provide a more comprehensive framework for future experimental validation [50]. The current findings should be interpreted as preliminary and hypothesis‐generating.

## 4. Conclusion

This study provides a comprehensive in silico evaluation of five major bioactive compounds identified in honey, namely, fumaric acid, p‐hydroxybenzoic acid, p‐coumaric acid, trans‐2‐hydroxycinnamic acid, and chrysin, with respect to their physicochemical characteristics, pharmacokinetic behavior, toxicity profiles, and potential interactions with inflammation‐related molecular targets. The results demonstrated that all investigated compounds possess favorable drug‐likeness characteristics, high predicted GI absorption, and generally low toxicity risks, supporting their potential suitability for further pharmacological development. Among the evaluated molecules, p‐coumaric acid and trans‐2‐hydroxycinnamic acid exhibited the most balanced combination of physicochemical and pharmacokinetic properties, whereas chrysin displayed the strongest lipophilic and receptor‐binding characteristics despite its lower aqueous solubility. Molecular docking analyses further revealed that trans‐2‐hydroxycinnamic acid and chrysin exhibited the most favorable binding affinities toward key inflammatory targets, including COX‐2, iNOS, NF‐κB, TNF‐α, IL‐6, and AKT1, suggesting their potential involvement in the modulation of multiple inflammatory signaling pathways. These findings are consistent with previous experimental evidence describing the anti‐inflammatory and antioxidant properties of honey‐derived phenolic compounds. The integrated ADME, toxicity, and docking results support the hypothesis that honey‐derived phenolics may contribute to the biological activities of honey through complementary and multitarget mechanisms involving the regulation of oxidative stress, inflammatory mediators, and intracellular signaling pathways. Such mechanisms may be particularly relevant for the development of novel complementary approaches targeting chronic inflammatory disorders. Nevertheless, the present findings are based exclusively on computational predictions and should therefore be interpreted with caution. Experimental validation through in vitro cellular assays, target‐specific enzyme inhibition studies, pharmacokinetic investigations, and in vivo disease models is required to confirm the predicted biological activities, molecular interactions, and safety profiles of these compounds. Future studies should also explore potential synergistic interactions among honey phenolics, as such interactions may contribute substantially to the therapeutic effects traditionally attributed to whole honey preparations.

Overall, the integrated experimental and computational findings indicate that honey‐derived phenolic compounds possess promising antioxidant and anti‐inflammatory properties, supported by favorable pharmacokinetic and safety profiles. Among the investigated compounds, trans‐2‐hydroxycinnamic acid, p‐coumaric acid, and chrysin emerged as the most promising candidates for further pharmacological investigation. Although the present findings provide valuable mechanistic insights, additional in vitro and in vivo studies are required to validate the predicted biological activities and therapeutic potential of these compounds in inflammatory diseases.

## Author Contributions

**Eda Sonmez Gurer**: conceptualization, data curation, methodology, software, formal analysis, visualization. **Sevgi Durna Dastan**: conceptualization, data curation, methodology, validation, writing – original draft, writing – review and editing. **Zeliha Selamoglu**: conceptualization, investigation, writing – original draft, writing – review and editing, supervision. **Ainash Oshibayeva**: investigation, project administration, funding acquisition, resources. **Taner Dastan**: methodology, software, validation, formal analysis, visualization. **Nurdana Salybekova**: investigation, supervision, funding acquisition, resources.

## Funding

This study was supported by the Project BR24992814 on the development of innovative technologies and creation of scientific infrastructure for the sustainable development of the South Kazakhstan region, funded by the Scientific Committee of the Ministry of Science and Higher Education of the Republic of Kazakhstan.

## Conflicts of Interest

The authors declare no conflicts of interest.

## Data Availability

All data generated or analyzed during this study are included in this published article. The SwissADME platform is freely accessible at http://www.swissadme.ch. Compound SMILES notations were retrieved from the PubChem database (https://pubchem.ncbi.nlm.nih.gov/).

## References

[1] Samarghandian S. , Farkhondeh T. , and Samini F. , Honey and health: A Review of Recent Clinical Research, Pharmacognosy Research. (2017) 9, 121–127. 28539734 10.4103/0974-8490.204647 PMC5424551

[2] Cianciosi D. , Forbes-Hernandez T. Y. , and Afrin S. , et al.Phenolic Compounds in Honey and Their Associated Health Benefits: A Review, Molecules. (2018) 23, no. 9, 10.3390/molecules23092322, 2322. 30208664 PMC6225430

[3] Alvarez-Suarez J. M. , Gasparrini M. , Forbes-Hernandez T. , Mazzoni L. , and Giampieri F. , The Composition and Biological Activity of Honey: A Focus on Manuka Honey, Foods. (2014) 3, no. 3, 420–432, 10.3390/foods3030420. 28234328 PMC5302252

[4] Ferreres F. , Tomás-Barberán F. A. , Soler C. , García-Viguera C. , Ortiz A. , and Tomás-Lorente F. , A Simple Extractive Technique for Honey Flavonoid HPLC Analysis, Apidologie. (1994) 25, no. 1, 21–30, 10.1051/apido:19940103.

[5] Can Z. , Yildiz O. , Sahin H. , Turumtay E. A. , Silici S. , and Kolayli S. , An Investigation of Turkish Honeys: Their Physico-Chemical Properties, Antioxidant Capacities and Phenolic Profiles, Food Chemistry. (2015) 180, 133–141, 10.1016/j.foodchem.2015.02.024. 25766810

[6] Kędzierska-Matysek M. , Stryjecka M. , Teter A. , Skałecki P. , Domaradzki P. , and Florek M. , Relationships Between the Content of Phenolic Compounds and the Antioxidant Activity of Polish Honey Varieties as a Tool for Botanical Discrimination, Molecules. (2021) 26, no. 6, 10.3390/molecules26061810, 26. PMC8004674 33806954

[7] Papi A. , Brightling C. , Pedersen S. E. , and Reddel H. K. , Asthma, The Lancet. (2018) 391, no. 10122, 783–800, 10.1016/S0140-6736(17)33311-1. 29273246

[8] Lambrecht B. N. and Hammad H. , The Immunology of Asthma, Nature Immunology. (2015) 16, no. 1, 45–56, 10.1038/ni.3049. 25521684

[9] Holgate S. T. , Wenzel S. , Postma D. S. , Weiss S. T. , Renz H. , and Sly P. D. , Asthma, Nature Reviews Disease Primers. (2015) 1, no. 1, 10.1038/nrdp.2015.25, 15025. PMC7096989 27189668

[10] Barnes P. J. , Targeting Cytokines to Treat Asthma and Chronic Obstructive Pulmonary Disease, Nature Reviews Immunology. (2018) 18, no. 7, 454–466, 10.1038/s41577-018-0006-6. 29626211

[11] Mims J. W. , Asthma: Definitions and Pathophysiology, International Forum of Allergy & Rhinology. (2015) 5, no. S1, S2–S6, 10.1002/alr.21609. 26335832

[12] Abbas A. S. , Ghozy S. , and Minh L. H. N. , et al.Honey in Bronchial Asthma: From Folk Tales to Scientific Facts, Journal of Medicinal Food. (2019) 22, no. 6, 543–550, 10.1089/jmf.2018.4303. 31135254

[13] Linker R. A. , Lee D.-H. , and Ryan S. , et al.Fumaric Acid Esters Exert Neuroprotective Effects in Neuroinflammation via Activation of the Nrf2 Antioxidant Pathway, Brain. (2011) 134, no. 3, 678–692, 10.1093/brain/awq386. 21354971

[14] Pei K. , Ou J. , Huang J. , and Ou S. , *p*-Coumaric Acid and Its Conjugates: Dietary Sources, Pharmacokinetic Properties and Biological Activities, Journal of the Science of Food and Agriculture. (2016) 96, no. 9, 2952–2962, 10.1002/jsfa.7578. 26692250

[15] Neog M. K. , Joshua Pragasam S. , Krishnan M. , and Rasool M. , *p*-Coumaric Acid, a Dietary Polyphenol Ameliorates Inflammation and Curtails Cartilage and Bone Erosion in the Rheumatoid Arthritis Rat Model, BioFactors. (2017) 43, no. 5, 698–717, 10.1002/biof.1377. 28742266

[16] Taofiq O. , González-Paramás A. , Barreiro M. , and Ferreira I. , Hydroxycinnamic Acids and Their Derivatives: Cosmeceutical Significance, Challenges and Future Perspectives, a Review, Molecules. (2017) 22, no. 2, 10.3390/molecules22020281, 281. 28208818 PMC6155946

[17] Yao J. , Jiang M. , Zhang Y. , Liu X. , Du Q. , and Feng G. , Chrysin Alleviates Allergic Inflammation and Airway Remodeling in a Murine Model of Chronic Asthma, International Immunopharmacology. (2016) 32, 24–31, 10.1016/j.intimp.2016.01.005. 26780233

[18] Bae Y. , Lee S. , and Kim S.-H. , Chrysin Suppresses Mast Cell-Mediated Allergic Inflammation: Involvement of Calcium, Caspase-1 and Nuclear Factor-κB, Toxicology and Applied Pharmacology. (2011) 254, no. 1, 56–64, 10.1016/j.taap.2011.04.008. 21515303

[19] Ongalbek D. , Tokul-Ölmez Ö. , and Şahin B. , et al.Classification of Buckwheat Honey Produced in Kazakhstan According to Their Biochemical Ingredients and Bioactivities by Chemometric Approach, Food Chemistry. (2024) 451, 10.1016/j.foodchem.2024.139409, 139409. 38692236

[20] Kamaruzaman N. A. , Sulaiman S. A. , Kaur G. , and Yahaya B. , Inhalation of Honey Reduces Airway Inflammation and Histopathological Changes in a Rabbit Model of Ovalbumin-Induced Chronic Asthma, BMC Complementary and Alternative Medicine. (2014) 14, no. 1, 10.1186/1472-6882-14-176, 176. 24886260 PMC4048365

[21] da Silva P. M. , Gauche C. , Gonzaga L. V. , Costa A. C. O. , and Fett R. , Honey: Chemical Composition, Stability and Authenticity, Food Chemistry. (2016) 196, 309–323, 10.1016/j.foodchem.2015.09.051. 26593496

[22] Ferreira L. L. G. and Andricopulo A. D. , ADMET Modeling Approaches in Drug Discovery, Drug Discovery Today. (2019) 24, no. 5, 1157–1165, 10.1016/j.drudis.2019.03.015. 30890362

[23] Daina A. , Michielin O. , and Zoete V. , SwissADME: A Free Web Tool to Evaluate Pharmacokinetics, Drug-Likeness and Medicinal Chemistry Friendliness of Small Molecules, Scientific Reports. (2017) 7, no. 1, 10.1038/srep42717, 42717. 28256516 PMC5335600

[24] Kim S. , Chen J. , and Cheng T. , et al.PubChem 2023 update, Nucleic Acids Research. (2023) 51, no. D1, D1373–D1380, 10.1093/nar/gkac956. 36305812 PMC9825602

[25] Daina A. and Zoete V. , A BOILED-Egg to Predict Gastrointestinal Absorption and Brain Penetration of Small Molecules, ChemMedChem. (2016) 11, no. 11, 1117–1121, 10.1002/cmdc.201600182. 27218427 PMC5089604

[26] Ghose A. K. , Viswanadhan V. N. , and Wendoloski J. J. , A Knowledge-Based Approach in Designing Combinatorial or Medicinal Chemistry Libraries for Drug Discovery. 1. A Qualitative and Quantitative Characterization of Known Drug Databases, Journal of Combinatorial Chemistry. (1999) 1, no. 1, 55–68, 10.1021/cc9800071. 10746014

[27] Hanwell M. D. , Curtis D. E. , Lonie D. C. , Vandermeersch T. , Zurek E. , and Hutchison G. R. , Avogadro: An Advanced Semantic Chemical Editor, Visualization, and Analysis Platform, Journal of Cheminformatics. (2012) 4, no. 1, 10.1186/1758-2946-4-17, 17. 22889332 PMC3542060

[28] The UniProt Consortium , UniProt: The Universal Protein Knowledgebase in 2023, Nucleic Acids Research. (2023) 51, no. D1, D523–D531, 10.1093/nar/gkac1052. 36408920 PMC9825514

[29] Burley S. K. , Bhikadiya C. , and Bi C. , et al.RCSB Protein Data Bank: Powerful New Tools for Exploring 3D Structures of Biological Macromolecules for Basic and Applied Research and Education in Fundamental Biology, Biomedicine, Biotechnology, and Disease, Nucleic Acids Research. (2021) 49, no. D1, D437–D451, 10.1093/nar/gkaa1038. 33211854 PMC7779003

[30] Jumper J. , Evans R. , and Pritzel A. , et al.Highly Accurate Protein Structure Prediction With AlphaFold, Nature. (2021) 596, no. 7873, 583–589, 10.1038/s41586-021-03819-2. 34265844 PMC8371605

[31] Liu Y. , Yang X. , Gan J. , Chen S. , Xiao Z.-X. , and Cao Y. , CB-Dock2: Improved Protein–Ligand Blind Docking by Integrating Cavity Detection, Docking and Homologous Template Fitting, Nucleic Acids Research. (2022) 50, no. W1, W159–W164, 10.1093/nar/gkac394. 35609983 PMC9252749

[32] Trott O. and Olson A. J. , AutoDock Vina: Improving the Speed and Accuracy of Docking With a New Scoring Function, Efficient Optimization, and Multithreading, Journal of Computational Chemistry. (2010) 31, no. 2, 455–461, 10.1002/jcc.21334. 19499576 PMC3041641

[33] Dassault Systèmes , BIOVIA Discovery Studio Visualizer (Sürüm 24.1.0), 2024, Dassault Systèmes.

[34] Bag G. C. , Grihanjali Devi P. , and Bhaigyaba T. , Assessment of Total Flavonoid Content and Antioxidant Activity of Methanolic Rhizome Extract of Three Hedychium Species of Manipur Valley, International Journal of Pharmaceutical Sciences Review and Research. (2015) 30, no. 1, 154–159.

[35] Singleton V. L. , Orthofer R. , and Lamuela-Raventós R. M. , Analysis of Total Phenols and Other Oxidation Substrates and Antioxidants by Means of Folin–Ciocalteu Reagent, Methods in Enzymology. (1999) 299, 152–178, 10.1016/S0076-6879(99)99017-1.

[36] Clarke G. , Ting K. , Wiart C. , and Fry J. , High Correlation of 2,2-diphenyl-1-picrylhydrazyl (DPPH) Radical Scavenging, Ferric Reducing Activity Potential and Total Phenolics Content Indicates Redundancy in use of All Three Assays to Screen for Antioxidant Activity of Extracts of Plants From the Malaysian Rainforest, Antioxidants. (2013) 2, no. 1, 1–10, 10.3390/antiox2010001. 26787618 PMC4665400

[37] Dharmadeva S. , Galgamuwa L. S. , Prasadinie C. , and Kumarasinghe N. , In Vitro Anti-Inflammatory Activity of *Ficus racemosa* L. Bark Using Albumin Denaturation Method, AYU. (2018) 39, no. 4, 239–242, 10.4103/ayu.AYU_27_18. 31367147 PMC6639822

[38] Lipinski C. A. , Lombardo F. , Dominy B. W. , and Feeney P. J. , Experimental and Computational Approaches to Estimate Solubility and Permeability in Drug Discovery and Development Settings, Advanced Drug Delivery Reviews. (2001) 46, no. 1–3, 3–26, 10.1016/S0169-409X(00)00129-0. 11259830

[39] Ritchie T. J. and Macdonald S. J. F. , The Impact of Aromatic ring Count on Compound Developability–Are Too Many Aromatic Rings a Liability in Drug Design?, Drug Discovery Today. (2009) 14, no. 21-22, 1011–1020, 10.1016/j.drudis.2009.07.014. 19729075

[40] Veber D. F. , Johnson S. R. , Cheng H.-Y. , Smith B. R. , Ward K. W. , and Kopple K. D. , Molecular Properties That Influence the Oral Bioavailability of Drug Candidates, Journal of Medicinal Chemistry. (2002) 45, no. 12, 2615–2623, 10.1021/jm020017n. 12036371

[41] Velika B. and Kron I. , Antioxidant Properties of Benzoic Acid Derivatives Against Superoxide Radical, Free Radicals and Antioxidants. (2012) 2, no. 4, 62–67, 10.5530/ax.2012.4.11.

[42] Arnott J. A. and Planey S. L. , The Influence of Lipophilicity in Drug Discovery and Design, Expert Opinion on Drug Discovery. (2012) 7, no. 10, 863–875, 10.1517/17460441.2012.714363. 22992175

[43] Delaney J. S. , ESOL: Estimating Aqueous Solubility Directly From Molecular Structure, Journal of Chemical Information and Computer Sciences. (2004) 44, no. 3, 1000–1005, 10.1021/ci034243x. 15154768

[44] Mani R. and Natesan V. , Chrysin: Sources, Beneficial Pharmacological Activities, and Molecular Mechanism of Action, Phytochemistry. (2018) 145, 187–196, 10.1016/j.phytochem.2017.09.016. 29161583

[45] Mohammadian F. , Pilehvar-Soltanahmadi Y. , Zarghami F. , Akbarzadeh A. , and Zarghami N. , Upregulation of miR-9 and Let-7a by Nanoencapsulated Chrysin in Gastric Cancer Cells, Artificial Cells, Nanomedicine, and Biotechnology. (2017) 45, no. 6, 1201–1206, 10.1080/21691401.2016.1216854. 27684298

[46] Schrödinger Release 2026-1 , LigPrep, 2025, Schrödinger, LLC.

[47] Schrödinger Release 2026-1 , Maestro, 2025, Schrödinger, LLC.

[48] Schrödinger Release 2026-1 , Protein Preparation Workflow, 2024, Schrödinger, LLC, Impact, Schrödinger, LLC, New York, NY; Prime, Schrödinger, LLC, New York, NY, 2025.

[49] Aller S. G. , Yu J. , and Ward A. , et al.Structure of P-Glycoprotein Reveals a Molecular Basis for Poly-Specific Drug Binding, Science. (2009) 323, no. 5922, 1718–1722, 10.1126/science.1168750. 19325113 PMC2720052

[50] Sliwoski G. , Kothiwale S. , Meiler J. , and Lowe E. W.Jr., Computational Methods in Drug Discovery, Pharmacological Reviews. (2014) 66, no. 1, 334–395, 10.1124/pr.112.007336. 24381236 PMC3880464

